# The Symptoms and Medications of Patients with Inflammatory Bowel Disease in Hubei Province after COVID-19 Epidemic

**DOI:** 10.1155/2020/2847316

**Published:** 2020-10-09

**Authors:** Huan Wang, Lei Tu, Ying Li, Tao Bai, Kaifang Zou, Fang Xiao, Jin Li, Min Chen, Heng Zhang, Gangqin Li, Yueyue Lu, Kai Wang, Shu Jin, Yuanping Yang, Liangru Zhu, Xiaohua Hou

**Affiliations:** ^1^Division of Gastroenterology, Union Hospital, Tongji Medical College, Huazhong University of Science and Technology, Wuhan, Hubei Province, China; ^2^Department of Gastroenterology, Tongji Hospital, Tongji Medical College, Huazhong University of Science and Technology, Wuhan, Hubei Province, China; ^3^Department of Gastroenterology, Zhongnan Hospital, Wuhan University, Wuhan, Hubei Province, China; ^4^Department of Gastroenterology, The Central Hospital of Wuhan, Wuhan, Hubei Province, China; ^5^Department of Gastroenterology, The Eighth Hospital of Wuhan, Wuhan, Hubei Province, China; ^6^Department of Gastroenterology, The First Affiliated Hospital of Changjiang University, Jingzhou, Hubei Province, China; ^7^Department of Gastroenterology, Jingzhou Hospital, Tongji Medical College, Huazhong University of Science and Technology, Jingzhou, Hubei Province, China; ^8^Department of Gastroenterology, Taihe Hospital, Huei University of medicine, Shiyan, Hubei Province, China; ^9^Department of Gastroenterology, Yichang Central Hospital, Yichang, Hubei, China

## Abstract

**Objectives:**

The COVID-19 epidemic triggered by coronavirus SARS-CoV-2 is rapidly spreading around the globe. This study is aimed at finding out the suspected or confirmed SARS-CoV-2 infection in patients with inflammatory bowel disease (IBD) in Hubei province, China. We also investigated symptoms, medications, life quality, and psychological issues of IBD patients under the ongoing pandemic.

**Methods:**

We conducted a self-reported questionnaire survey via an online survey platform. SARS-CoV-2 infection-related data was collected from IBD patients. The status quo of medications and symptoms of the subjects were investigated. Life quality, depression, and anxiety were measured by clinical questionnaires and rated on scoring systems.

**Results:**

A total of 204 IBD patients from Hubei province were included in this study. No suspected or confirmed SARS-CoV-2 infection case was found in this study. As a result of city shutdown, two-thirds of the patients (138/204) in our series reported difficulty in accessing medicines and nearly half of them (73/138) had to discontinue medications. Apart from gastrointestinal symptoms, systemic symptoms were common while respiratory symptoms were rare in the cohort. Though their quality of life was not significantly lowered, depression and anxiety were problems that seriously affected them during the COVID-19 epidemic.

**Conclusions:**

Inaccessibility to medications is a serious problem for IBD patients after city shutdown. Efforts have to be made to address the problems of drug withdrawal and psychological issues that IBD patients suffer from during the COVID-19 outbreak.

## 1. Introduction

Coronavirus disease 2019 (COVID-19), triggered by severe acute respiratory syndrome coronavirus 2 (SARS-CoV-2), hit Hubei province at the end of 2019 and has turned into a global pandemic [1]. The cases of COVID-19 have been soaring around the globe over the past months. As of June 7th, 2020, 6 896 800 cases have been reportedly confirmed and 397 588 have died in over 200 countries [2].

Both experimental and clinical data showed that people of all ages can fall victim to SARS-CoV-2, especially elderly adults and those with underlying diseases. Inflammatory bowel disease (IBD) is an idiopathic inflammatory condition involving gastrointestinal tract. The use of glucocorticoids, immunosuppressants, and other immunity-weakening medications in IBD patients may increase the risk of opportunistic infections [3]. Surveillance Epidemiology of Coronavirus Under Research Exclusion (SECURE-IBD) reported that 1379 IBD patients were complicated with COVID-19 as of June 7th, mainly from Europe and America but only one case in China [4]. In this study, we conducted a questionnaire survey with an attempt to obtain accurate data concerning the *status quo* of SARS-CoV-2 infection in IBD patients in Hubei.

Naturally, IBD patients alternately go through periods of remission and relapses that entail regular medication and follow-up. However, in areas hit hard by COVID-19, lockdown and travel restrictions are introduced. As a consequence, IBD patients may not be followed up as scheduled and may have limited or no access to medicines they need. The disease may relapse or deteriorate if patients are cut off from their medications. We believe that the data regarding of the current situation of IBD patients is of great value to doctors responsible for the management of IBD patients under the epidemic of COVID-19.

The mounting confirmed cases of COVID-19 infections and deaths, as reported by various media, are posing tremendous psychological pressure on IBD patients. In fact, IBD patients were reported to be associated with mental stress and anxiety [5]. Psychological stress has been identified as a worsening factor for IBD. Adverse life events and chronically perceived stress may contribute to elevated anxiety, aggravation of inflammation, and disease relapse in IBD patients [6]. Therefore, understanding the mental status of IBD patients during COVID-19 epidemic can help physicians take measures to reduce the impact of negative emotions on the patients.

This study comprehensively investigated the current status of IBD patients during the epidemic of COVID-19, including symptoms that may or may not be related to COVID-19, medications, mental health state, and quality of life, with an attempt to help doctors understand the physical and mental conditions of IBD patients and better manage IBD patients under the ongoing pandemic.

## 2. Methods

### 2.1. Study Participants

This study included 204 IBD patients admitted to level-A and class one hospitals in Hubei province, China. IBD was diagnosed on the basis of conventional criteria of the disease. All patients were electronically invited to anonymously complete an online questionnaire from February 7th to March 15th, 2020, during the COVID-19 epidemic.

### 2.2. Study Design

The clinical study was of observational and cross-sectional nature. A self-reported questionnaire survey was conducted via an online survey platform (“SurveyStar,” Changsha Ranxing Science and Technology, Shanghai, China). Demographic and social data were collected from IBD patients. Quality of life depression and anxiety were rated against validated clinical questionnaires and scoring systems. This study was approved by the Medical Ethical Review Committee, Union Hospital, Tongji Medical College, Huazhong University of Science and Technology, Wuhan, China ([2020] No. 0033).

### 2.3. Questionnaires

Sociodemographic information of IBD patients mainly included age, gender, body weight, education background, marital status, education, occupations, place of residence during the COVID-19 epidemic, and smoking status (smoker and nonsmoker/past smoker).

IBD-related data included subtypes, course, and activity of the condition. The data on treatments of the patients received before the COVID-19 epidemic were also harvested. Digestive symptoms, such as abdominal pain, diarrhea, bloody stools, perianal discomfort, and vomiting were also involved. Due to the home quarantine imposed in most regions, the subjects did not have full access to medicines. Data on drug withdrawal and symptom changes were also collected.

In order to identify the cases suspected of SARS-CoV-2 infection, we used a recent guideline of diagnosis and treatment of COVID-19 (version7) [7], released by the National Health Commission in China. A suspected COVID-19 patient was defined as someone fulfilling both of the following criteria: (1) having at least one of the following symptoms or signs, including fever, respiratory symptoms, such as cough, sore throat, or dyspnea or radiographic evidence of pneumonia, and reduced counts of white blood cell or lymphocytes and (2) recently having lived in or paid visit to or gone to cities where community transmission of SARS-CoV-2 virus was reported, including Wuhan city, or recently having close contact with a confirmed COVID-19 patient or patients with fever/respiratory symptoms from Wuhan within 14 days before illness onset.

A confirmed case was defined as a patient testing positive for SARS-CoV-2 by PCR detection of upper respiratory specimen or for IgM and IgG in the serum. We harvested data on contact history, clinical symptoms, and examination results in the past two weeks. In particular, respiratory symptoms mainly included stuffy nose, nasal discharge, cough, sputum production, and chest pain. Systemic symptoms involved fever, fatigue, debility, muscle pain, headache, and palpitations.

Inflammatory Bowel Disease Questionnaire (IBDQ) is a 32-item, disease-specific health-related quality of life (HRQoL) questionnaire [8]. It consists of 4 domains, i.e., bowel symptoms, systemic symptoms, emotional function, and social function. Responses to each item are scored on a 7-point scale, in which 1 was listed as worst and 7 the best. The total IBDQ points range from 32 to 224, with a higher score indicative of better quality of life [9].

Depression was assessed against the Patient Health Questionnaire (PHQ-9). The PHQ-9 contains nine items that assess, from 9 aspects, if a major depression is present [10]. The total score ranges from 0 to 27 points. It rates depression on four different levels: mild, moderate, moderately severe, and severe, on the basis of cut-off scores of 5, 10, 15, and 20, respectively [11]. 10 was the cut-off score for depression status.

Anxiety was measured by the Generalized Anxiety Disorder Assessment (GAD-7) [12]. GAD-7 includes 7 items, and each is scored in terms of frequency on a scale from 0 to 3. The total scores range from 0 to 21. It categorizes anxiety into three degrees: mild, moderate, and severe, on the basis of cut-off scores of 5, 10, and 15.

### 2.4. Statistical Analysis

Data was presented as the mean ± standard deviation (SD). Differences between groups were evaluated by using *t*-test or analysis of variance (ANOVA). Pearson's correlation analysis and multivariate analysis were employed to determine the structural relationship between the measured variables. Statistical analysis was performed using SPSS statistical package (version 17).

## 3. Results

### 3.1. Subjects

A total of 204 IBD patients were found in Hubei province, and they were aged 35.14 ± 11.50 years, with a BMI of 22.57 ± 8.01 kg/m^2^ on average. There were 123 men and 81 women. 41 patients were from Wuhan, and 163 from other cities of Hubei province (Table 1).

### 3.2. Status Quo of IBD Patients

In our series, 116 patients had Crohn's disease (CD); 85 had ulcerative colitis (UC); and 3 had undefined IBD (IBDU). 43.14% (88/204) of the IBD patients remained in remission while 26.47% (54/204) of them were in active stage. The other 62 patients were uncertain of their disease activity. The main drugs the IBD patients took were aminosalicylic acid in 48.53% (99/204), glucocorticoid in 9.31% (19/204), immunosuppressants in 22.55% (46/204), biological agents in 31.37% (64/204), and enteral nutrition drugs in 11.76% (24/204).

### 3.3. Inaccessibility to Medications as a Serious Problem for IBD Patients

**Figure 1:**
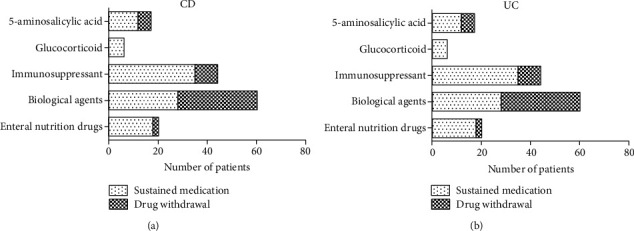
Continuation and discontinuation of medications in IBD patients during the COVID-19 epidemic. (a) The number of CD patients who continued and discontinued their medications (5-aminosalicylic acid, glucocorticoid, immunosuppressants, biological agents, and enteral nutrition drugs). (b) The number of UC patients who continued and discontinued their medications.

More than two-thirds of patients (138/204) reported difficulty in accessing medicines as a consequence of city shutdown during the COVID-19 epidemic. Drug withdrawal occurred in half of them (70/138). Biological agents, a major medication used by CD patients, topped the list as a drug hard to procure. Half of the patients (32/60) discontinued the biological agents they had been on. Came next are immunosuppressants and 5-aminosalicylic acid (Figure 1(a)). In UC patients, 23.26% (20/86) of patients had to stop taking 5-aminosalicylic acid (Figure 1(b)).

54.29% (38/70) of patients who discontinued medication reported recurrence and exacerbation of symptoms, such as abdominal pain (71.05%), diarrhea (50%), bloody stools (31.58%), perianal discomfort (21.05%), and vomiting (10.53%)

### 3.4. Gastrointestinal, Respiratory, and Systemic Symptoms in IBD Patients

More than half of patients (106/204) with IBD had gastrointestinal symptoms in the last two weeks during the COVID-19 epidemic. Abdominal pain (24.51%) and diarrhea (25.00%) remained predominant symptoms, followed by perianal discomfort (16.18%) and bloody stools (15.69%). Nausea and vomiting (3.43%) were extremely rare.

A few IBD patients (8.82%) reported respiratory symptoms in the last two weeks, including stuffy nose (4.90%), nasal discharge (3.92%), cough (1.96%), sputum production (1.47%), and chest pain (0.98%).

Constitutional or systemic symptoms were very common in patients with IBD, which could easily be confused with SARS-CoV-2 infection. 26.24% of the patients had general symptoms in the last two weeks, such as listlessness (16.29%), fatigue (14.03%), muscle pain (6.79%), headache (4.52%), and palpitations (2.71%). Nine patients developed fever, which was mostly mild or moderate (Table 2).

We used diagnostic criteria of COVID-19, as a reference, in combination with epidemiological data and clinical manifestations. No suspected SARS-CoV-2 infection case was found in our cohort. In this study, four patients had a definite contact history with the confirmed cases of COVID-19. One of them had mild fever, and the highest body temperature was lower than 38°C, presenting no respiratory symptoms. Two other patients had cough and sputum production. The last one only had listlessness, headache, and diarrhea, but without fever or respiratory symptoms. However, the four patients did not seek medical attention or receive any SARS-CoV-2-related tests or examinations. Besides, there were nine patients with mild or moderate fever and most of them were free of respiratory symptoms.

### 3.5. HRQoL Scores in IBD Patients during COVID-19 Epidemic

In order to find out the change in quality of life in IBD patients during the COVID-19 epidemic, we collected IBDQ score data that had been collected before the outbreak of COVID-19, from Jan 1st to Oct 31st, 2019. In CD patients, the total score after COVID-19 outbreak (171.16 ± 33.80, *n* = 113) was not significantly different from that before the epidemic (163.73 ± 30.77, *n* = 73). Moreover, the IBDQ scores in UC patients were similar to those in CD patients. The total score after COVID-19 outbreak (166.07 ± 37.87, *n* = 85) was not significantly different from that before the epidemic (153.36 ± 36.78, *n* = 33).

### 3.6. PHQ-9 and GAD-7 Scores of IBD Patients during COVID-19 Outbreak

Depression could be assessed for 201 patients and anxiety for 203 patients, and missing data is due to lack of or incomplete questionnaires. In our series, 21.39% (43/201) patients scored over 10 points and were considered to have depression. Furthermore, two patients suffered from severe depression. 22.39% (45/201) of them reported borderline depression symptoms (Table 3). 30.54% (62/203) of the IBD patients were found to have anxiety of various degrees. 21.57% (44/203) of the patients were mildly anxious. A few patients (7.35%) developed moderate anxiety. Three patients were considered to suffer from severe anxiety. The level of depression and anxiety between CD and UC seemed similar (Table 3).

### 3.7. Factors Associated with the Quality of Life, Depression, and Anxiety

The quality of life was found to be associated with the disease activity, travel restraint, or restriction and drug withdrawal during COVID-19 epidemic, but not with gender, marital status, smoking, occupations, and subtypes of IBD.

The level of depression and anxiety bore a correlation with education, disease activity, and travel restriction. Besides, mental workers (white-collar workers) were more anxious than their manual and mental-physical counterparts (Table 4).

## 4. Discussion

In this study, all of IBD patients were from Hubei province, the so-called “epicenter” of the COVID-19 outbreak. As a result of city shutdown, two-thirds of the patients in our series reported difficulty in accessing medications and drug discontinuation became a serious problem in patients with IBD. Apart from gastrointestinal symptoms, systemic symptoms were common in IBD patients while respiratory symptoms were rare. However, no suspected or confirmed SARS-CoV-2 infection cases were found in our series. Though the quality of life was not significantly decreased, depression and anxiety were serious problems that plagued the patients during the COVID-19 epidemic.

IBD is considered to be a group of immune disorders characterized by chronic gut inflammation [13, 14]. Since IBD patients were usually on biologics and immunosuppressive agents, they appeared to be vulnerable to serious and/or opportunistic infections [3] by such viruses as cytomegalovirus (CMV), Epstein-Barr virus (EBV), and herpes simplex virus (HSV) [15]. However, no increased risk of developing SARS-CoV-2 infection in IBD patients had been previously reported by other researchers [16]. In this study, no suspected SARS-CoV-2 infection case was found since the outbreak of the COVID-19 epidemic in Hubei province. Moreover, no IBD patient with SARS-CoV-2 infection has been reported from the tertiary IBD centers in Wuhan. Until now, only one patient was reportedly infected with SARS-CoV-2 in China [4]. However, in the United States, Spain, France, and Italy, in all there were more than nine hundred IBD patients suffering SARS-CoV-2 infection [4]. Many factors might be attributed to the discrepancy in the incidence rate of SARS-CoV-2 infection in IBD patients at home and abroad. Firstly, the incidence of IBD in China was considerably lower than in European and American. Second, the sample might be relatively small in our study. More importantly, several guidelines for managing IBD were released by the Chinese IBD Society in early February 2020 [17] and they covered home quarantine, dieting, personal protection, use of immunosuppressive agents, biologics, and cytokine blockers under IBD doctor's suggestions to prevent COVID-19 [16]. After completion of this survey, one UC patient coming to our hospital was found to be positive for IgG to while negative for IgM to SARS-CoV-2 before IFX injection; although SARS-CoV-2 RNA test showed negative, the positive for IgG demonstrated that he was previously infected with the virus. This patient had a fever of 38°C a month and half ago, but without respiratory symptoms. As a result, he did not seek medical attention or receive any SARS-Cov-2-related tests. Without special treatment, his symptoms subsided spontaneously after the home quarantine.

Gastrointestinal (GI) symptoms had been previously reported in a significant portion of COVID-19 patients, including diarrhea, nausea, vomiting, and abdominal pain [18–20]. Moreover, diarrhea was reportedly the most common GI symptom [21]. GI symptoms could even be the initial clinical manifestation of COVID-19. A study reported that 23.3% of mild COVID-19 patients did not present with respiratory symptoms but had only GI symptoms [22]. In our study, more than half of the IBD patients had GI symptoms in the last two weeks during the COVID-19 epidemic, especially abdominal pain and diarrhea. On the other hand, systemic symptoms, even fever, were very common in patients with IBD in our series. A few of them also developed respiratory symptoms that might be confused with COIVID-19. Therefore, IBD patients, particularly those in Hubei province, were extremely worried about SARS-CoV-2 infection and tended to seek medical attention, which might substantially increase their risk of infection. Given this, since Jan 29, 2020, China Crohn's & Colitis Foundation enlisted a great many IBD specialists, as volunteers, to offer online consultancy to IBD patients. Moreover, some IBD centers set up online IBD clinics. All these measures greatly avoided unnecessary visits to hospital or clinic and thereby minimized the risk of SARS-CoV-2 infection in IBD patients during the epidemic of COVID-19.

Duration of treatments is crucial to the management of IBD patients. In our study, two-thirds of the patients reported difficulty in accessing medicines and half of them discontinued their medications. A significant portion of patients reported recurrence and exacerbation of symptoms after drug withdrawal. Two IBD patients admitted to our hospital developed severe complications, including intestinal obstruction and perforation after drug withdrawal. It is extremely difficult for doctors to manage IBD patients during the city shutdown and under travel ban. We advise that all patients should continue their medications and avoid drug withdrawal whenever possible. Mesalamine and biologic agent treatments are important, and dose reduction is risky [23]. If intravenous infusion is not feasible, patients could use adalimumab as an alternative for infliximab since adalimumab allows for subcutaneous injection. However, any change in medication must be made in strict accordance with the directions of IBD specialists. New assessments, including self-reported questionnaire, online consultancy, and treatment are recommended [24]. Travel restrictions and city shutdown have forced IBD units and IBD patients to drastically change and restructure the way they handle IBD.

Our study showed that the quality of life in IBD patients was not significantly impacted after the outbreak of COVID-19. This might largely be ascribed to the relatively small sample size of this study. The strengths of the study are its relatively large sample size, and that it was conducted in the early phases of the outbreak. Moreover, the network questionnaire approach we used might produce, to some extent, information bias. Cases of IBD patients with less education or older age are less likely to participate in the network research. Therefore, cohort studies with larger samples are warranted to further investigate the change in the quality of life in IBD patients.

We found that the COVID-19 epidemic heavily inflicted depression and anxiety on the IBD patients in our series. The findings showed that 22.39% of the patients were considered to be depressed, and 30.54% of the patients experienced anxiety of varying degrees. A previous survey showed the prevalence of anxiety (21.2%) and depression (25.8%) in IBD patients by the same questionnaire of PHQ-9 and GAD-7 [25]. The prevalence of anxiety and depression during the COVID-19 epidemic in our study appeared higher than the previous, but the variation in the rates of depression and anxiety was likely due to differences in populations studied, methods used to evaluate depression and anxiety, and study designs. Nevertheless, the high prevalence of mental disorders found in our study is an important problem that requires further attention. IBD units and IBD specialists should pay more attention to the psychological issues of IBD patients during the COVID-19 epidemic. Furthermore, patient education, psychological counseling, and appropriate mental health support should be given. Antidepressant and antianxiety drugs could be applied to the patients with severe mental disorders whenever appropriate.

## 5. Conclusions

Inaccessibility to medications and status of depression and anxiety are serious problems in IBD patients after COVID-19 epidemic. This study helps doctors understand the physical and mental conditions of IBD patients and better manage IBD patients under the ongoing pandemic.

## Figures and Tables

**Table 1:** Characteristics of the patients with IBD responding to the questionnaire.

Numbers of patients, *n* (%)	204 (100%)
Gender, *n* (%)	
Male	123 (60.29%)
Female	81 (29.71%)
Age (mean ± SD)	35.14 ± 11.50
BMI (mean ± SD), kg/m^2^	22.57 ± 8.01
Time since diagnosis (mean ± SD)	3.93 ± 3.40
Habitat, *n* (%)	
Wuhan	41 (20.10%)
Outside of Wuhan in Hubei province	163 (79.90%)
Smoking, *n* (%)	
Nonsmoker	163 (79.90%)
Current smoker	12 (5.88%)
Past smoker	29 (14.22%)
Marital status, *n* (%)	
Unmarried	70 (34.31%)
Married	130 (63.73%)
Divorced	4 (1.96%)
Education level, *n* (%)	
Primary education	109 (53.43%)
University educated	95 (46.57%)
Occupation, *n* (%)	
Mental worker	62 (30.39%)
Manual worker	30 (14.71%)
Mental-physical worker	48 (23.53%)
Unoccupied	64 (31.37%)
Diagnosis, *n* (%)	
Ulcerative colitis	85 (41.67%)
Crohn's disease	116 (56.86%)
Inflammatory bowel disease unclassified	3 (1.47%)
Disease activity, *n* (%)	
Active stage	54 (26.47%)
Inactive stage	150 (73.53%)

**Table 2:** Gastrointestinal, respiratory, and systemic symptoms in IBD patients.

	CD	UC	IBDU	Total	%
Gastrointestinal symptoms					
Abdominal pain	28	22	0	50	24.51%
Diarrhea	29	22	0	51	25.00%
Nausea and vomiting	3	4	0	7	3.43%
Bloody stools	4	28	0	32	15.69%
Perianal discomfort	16	17	0	33	16.18%
Total	54	52	0	106	51.96%
Systemic symptoms					
Fever	6	4	0	10	4.90%
Fatigue	18	12	0	30	14.71%
Listlessness	19	16	0	35	17.16%
Headache	4	5	0	9	4.41%
Muscle pain	8	7	0	15	7.35%
Palpitations	2	4	0	6	2.94%
Total	30	26	0	56	27.45%
Respiratory symptoms					
Stuffy nose	5	4	1	10	8.82%
Nasal discharge	5	2	1	8	4.90%
Cough	2	2	0	4	3.92%
Sputum production	2	1	0	3	1.96%
Chest pain	0	1	1	2	1.47%
Total	11	5	2	18	8.82%

The numbers of patients with gastrointestinal, respiratory, or systemic symptoms were showed in different subtypes of IBD. Total numbers and percentage were displayed in the last two columns.

**Table 3:** Psychological status in IBD patients during the COVID-19 epidemic.

	IBD		CD		UC	
	*n*	%	*n*	%	*n*	%
Level of depression						
No depression	113	56.22%	67	57.76%	45	52.94%
Mild depression	45	22.39%	23	19.83%	21	24.71%
Moderate depression	27	13.43%	16	13.79%	11	12.94%
Moderately severe depression	14	6.97%	9	7.76%	5	5.89%
Moderately severe depression	2	1.00%	0	0.00%	2	2.35%
Level of anxiety						
No anxiety	141	69.12%	84	72.41%	54	63.53%
Mild anxiety	44	21.57%	24	20.69%	20	23.53%
Moderate anxiety	15	7.35%	6	5.17%	9	10.59%
Severe anxiety	3	1.47%	2	1.72%	1	1.18%

The numbers and percentage of different levels of depression and anxiety were displayed in IBD patients.

**Table 4:** The multiple-factor analysis of the quality of life, depression, and anxiety.

	IBDQ		Depression		Anxiety	
	AR^2^	*p*	AR^2^	*p*	AR^2^	*p*
Gender	-0.001	0.408	-0.004	0.852	-0.005	0.983
BMI	0.01	0.071	0.02	0.022^∗^	0.015	0.913
Smoking	-0.003	0.557	-0.005	0.956	-0.001	0.365
Marital status	0	0.325	0.006	0.125	-0.004	0.898
Education	0.025	0.011^∗^	0.011	0.065	0.033	0.004^∗∗^
Occupation	-0.001	0.38	0.014	0.044^∗^	0.031	0.005^∗∗^
Subtypes of IBD	0	0.312	-0.004	0.7	0.001	0.281
Disease activity	0.008	<0.001^∗^	0.058	<0.001∗∗	0.062	<0.001∗∗
Residence area	-0.005	0.988	-0.003	0.589	0.006	0.133
Traffic restraint	0.044	0.001^∗∗^	0.028	0.008^∗∗^	0.015	0.038^∗^
Drug withdrawal	0.032	0.016^∗^	0.012	0.097	0	0.322

^∗^ *p* < 0.05; ^∗∗^*p* < 0.01.

## Data Availability

The data used to support the findings of this study are included within the article. The data also are available from the corresponding author upon request.
